# Acid tolerance in early colonizers of oral biofilms

**DOI:** 10.1186/s12866-021-02089-2

**Published:** 2021-02-14

**Authors:** Gabriella Boisen, Julia R. Davies, Jessica Neilands

**Affiliations:** Section for Oral Biology and Pathology, Faculty of Odontology, and Biofilms - Research Center for Biointerfaces, Malmö University, SE-205 06 Malmö, Sweden

**Keywords:** Streptococci, *Actinomyces*, Salivary proteins, Pellicle, Acid tolerance response

## Abstract

**Background:**

In caries, low pH drives selection and enrichment of acidogenic and aciduric bacteria in oral biofilms, and development of acid tolerance in early colonizers is thought to play a key role in this shift. Since previous studies have focussed on planktonic cells, the effect of biofilm growth as well as the role of a salivary pellicle on this process is largely unknown. We explored acid tolerance and acid tolerance response (ATR) induction in biofilm cells of both clinical and laboratory strains of three oral streptococcal species (*Streptococcus gordonii*, *Streptococcus oralis and Streptococcus mutans*) as well as two oral species of *Actinomyces (A. naeslundii and A. odontolyticus)* and examined the role of salivary proteins in acid tolerance development.

**Methods:**

Biofilms were formed on surfaces in Ibidi® mini flow cells with or without a coating of salivary proteins and acid tolerance assessed by exposing them to a challenge known to kill non-acid tolerant cells (pH 3.5 for 30 min) followed by staining with LIVE/DEAD BacLight and confocal scanning laser microscopy. The ability to induce an ATR was assessed by exposing the biofilms to an adaptation pH (pH 5.5) for 2 hours prior to the low pH challenge.

**Results:**

Biofilm formation significantly increased acid tolerance in all the clinical streptococcal strains (*P* < 0.05) whereas the laboratory strains varied in their response. In biofilms, *S. oralis* was much more acid tolerant than *S. gordonii* or *S. mutans. A. naeslundii* showed a significant increase in acid tolerance in biofilms compared to planktonic cells (*P* < 0.001) which was not seen for *A. odontolyticus*. All strains except *S. oralis* induced an ATR after pre-exposure to pH 5.5 (*P* < 0.05). The presence of a salivary pellicle enhanced both acid tolerance development and ATR induction in *S. gordonii* biofilms (*P* < 0.05) but did not affect the other bacteria to the same extent.

**Conclusions:**

These findings suggest that factors such as surface contact, the presence of a salivary pellicle and sensing of environmental pH can contribute to the development of high levels of acid tolerance amongst early colonizers in oral biofilms which may be important in the initiation of caries.

## Background

The human oral microbiota comprises more than 600 different bacterial species of which around 100–200 can be found in a single individual [1]. Normally the microbiota is dominated by species belonging to the genera *Streptococcus* and *Actinomyces* as well as *Neisseria*, *Veillonella* and *Granulicatella* [2]. Bacteria colonizing the oral cavity are mainly found as complex, multispecies biofilms attached to the hard and soft-tissue surfaces. On the teeth, biofilm formation is initiated by interactions between bacteria and the acquired enamel pellicle, a thin film of proteins derived from saliva and gingival crevicular fluid [3]. Early colonizers such as *Streptococcus* and *Actinomyces*, can adhere to the tooth surface through non-specific interactions as well as specific binding of bacterial surface adhesins to salivary proteins within the pellicle [4, 5]. As biofilms mature, diversity increases due to co-aggregation of new species to previously adhered bacteria and modification of the environment to extend the range of ecological niches [6]. Under normal conditions, the primary nutrient source for bacteria in supra-gingival biofilms is saliva, and its component proteins and glycoproteins, can be co-operatively degraded by members of the biofilm community [7, 8]. However, in response to the intermittent intake of dietary carbohydrates, which overwhelms the bacteria with a rich source of fermentable sugars, species such as streptococci and *Actinomyces* produce lactic acid, resulting in a fall in biofilm pH [9]. In health, compensatory mechanisms, including buffering by saliva and the generation of alkaline end-products from salivary urea and arginine by bacteria, are able to counteract this perturbation and homeostasis is re-established [10]. This has been termed the ‘dynamic stability stage’ by Nyvad and Takahashi [11] and under these conditions, the composition of the community remains relatively stable. However, when severe ecological stresses overcome the resilience of the biofilm, the balance is perturbed leading to changes in biofilm physiology and composition [12]. According to the ‘ecological plaque hypothesis’, frequent episodes of low biofilm pH drive the selection of an acid tolerant microbiota that can continue to metabolize and produce acid even when the pH is low [13]. This corresponds to the ‘acidogenic stage’ as described by Nyvad and Takahashi [11]. The increased duration of low pH pushes the de/re-mineralization balance towards net dissolution of the oral hard tissues, eventually resulting in the development of caries lesions. Dental caries is thought to affect around 2.5 billion people worldwide [14] and estimates suggest that around 5% of the healthcare budgets of the Organisation for Economic Co-operation and Development (OECD) countries are consumed by the disease [15].

*Streptococcus mutans* has long been considered as an important agent in caries development largely due to its isolation from carious sites and its ability to withstand low pH [16]. However, since *S. mutans* can be present at low levels, or even absent, in caries lesions and individuals with *S. mutans* do not always have caries, the presence of this bacteria is more likely to be an indicator of low pH conditions in the biofilm rather than the causative agent [17–19]. Therefore, other prominent members of oral biofilms *i.e* various species of non-mutans streptococci including *Streptococcus sanguinis*, *Streptococcus oralis*, *Streptococcus gordonii* and *Streptococcus mitis* as well as *Actinomyces spp* must play a significant role in driving the development of an acid tolerant microbiota that initiates the development of caries [20]. Experiments in planktonic culture have shown that some of these bacteria are able to adapt and survive under low pH conditions and the development of acid tolerance has been attributed to their capacity to induce an acid tolerance response (ATR) when exposed to pH 5.5 [21, 22]. This response protects bacteria from intracellular low pH which denatures proteins and inhibits metabolic processes. The mechanisms underlying the development of an ATR have been widely investigated in *S. mutans* and have been shown to include a decrease in membrane permeability to protons and an increase in proton expulsion from the cells, synthesis of a range of chaperones that protect proteins and nucleic acids from acid denaturation and enzymatic DNA-repair [23]. In contrast, acid tolerance development in non-mutans streptococci and other oral species has not been as well studied, although changes in fatty acid composition of the membrane, as well as activation of the arginine deiminase system have been described in *S. gordonii* and *S. sanguinis,* respectively in response to low pH [24, 25].

Biofilm growth has been shown to have profound effects on the physiological properties of bacteria, and characteristics such as sensitivity to antimicrobial agents and growth rates differ significantly between biofilm bacteria and their planktonic counterparts [26]. For example, *S. mutans* becomes more acid tolerant when grown in a biofilm as compared to planktonic growth [27, 28]. In this study we aimed to investigate the role of biofilm formation on development of acid tolerance and ATR induction in clinical and laboratory strains of early oral colonizers (non-mutans streptococci and *Actinomyces)* and to explore the effect of salivary proteins on these processes.

## Results

### Effect of early biofilm formation on acid tolerance

**Fig. 1:**
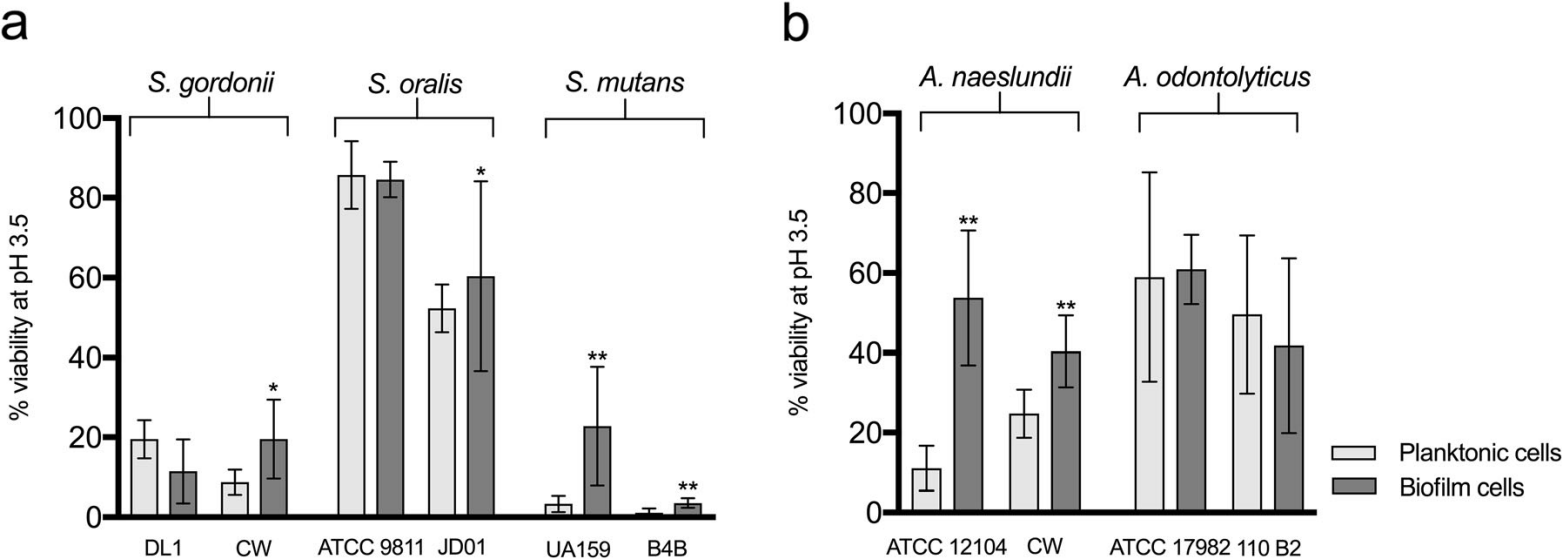
Comparison of the acid tolerance of different streptococcal (**a**) and *Actinomyces* (**b**) strains in planktonic culture and biofilms on uncoated surfaces. Acid tolerance was evaluated after an acid challenge (pH 3.5) followed by LIVE/DEAD® BacLight™ staining and CLSM, and expressed as % viability. The graphs show the mean and standard deviation of three independent biological replicates. *P < 0.05, **P < 0.001

The effect of biofilm formation on the acid tolerance of different early oral colonizing bacteria was assessed by determining the level of survival following a low pH challenge for cells adhered to an uncoated surface and comparing with their planktonic counterparts. No differences in the level of surface coverage of biofilm cells were seen between the biofilm control cells (kept at pH 7.5) and those exposed to the acid challenge used to differentiate acid tolerant from non-acid tolerant cells [i.e. exposure to acid did not remove the cells from the surface (data not shown)]. The clinical strains of *S. gordonii* (CW), *S. mutans* (B4B), and *S. oralis* (JD01) showed small but significant increases in acid tolerance in the biofilms compared to planktonic culture (2.1-fold, 4-fold, and 1.1-fold increase respectively, *P* < 0.05, Fig. 1a). For the laboratory strains, the picture was variable, with the *S. mutans* strain (UA159) showing a significant increase in acid tolerance in the biofilm (7.7-fold, *P* < 0.001) while no increase was seen for either *S. gordonii* (DL1) or *S. oralis* (ATCC9811). For all species, with the exception of *S. gordonii,* the biofilm acid tolerance of the laboratory strain was greater than that of the clinical one, although the differences were generally small and not significant. Comparison between the different streptococci showed that both the clinical and laboratory strains of *S. oralis* had much higher levels of biofilm acid tolerance than the *S. gordonii* and *S. mutans* strains (Fig. 1a). Interestingly, the clinical strain of *S. mutans* (B4B) showed the lowest acid tolerance of all the strains tested.

Both the clinical and the laboratory strain of *A. naeslundii* showed significantly greater acid tolerance in biofilms compared to their planktonic counterparts (*P* < 0.001, Fig. 1b), although the most pronounced increase was seen for *A. naeslundii* ATCC 12104, with a 4.9-fold increase compared with a 1.6-fold increase for *A. naeslundii* CW. In contrast, for the two *A. odontolyticus* strains, there was no increase in acid tolerance on biofilm formation. The overall levels of acid tolerance in biofilms were quite similar for *A. odontolyticus* and *A. naeslundii* (Fig. 1b). Thus, for the bacteria with a relatively high degree of acid tolerance in planktonic culture, biofilm formation had little or no additive effect whereas varying degrees of increased acid tolerance were seen for the other strains after biofilm formation.

### Effect of adherence to a salivary pellicle on acid tolerance

**Fig. 2:**
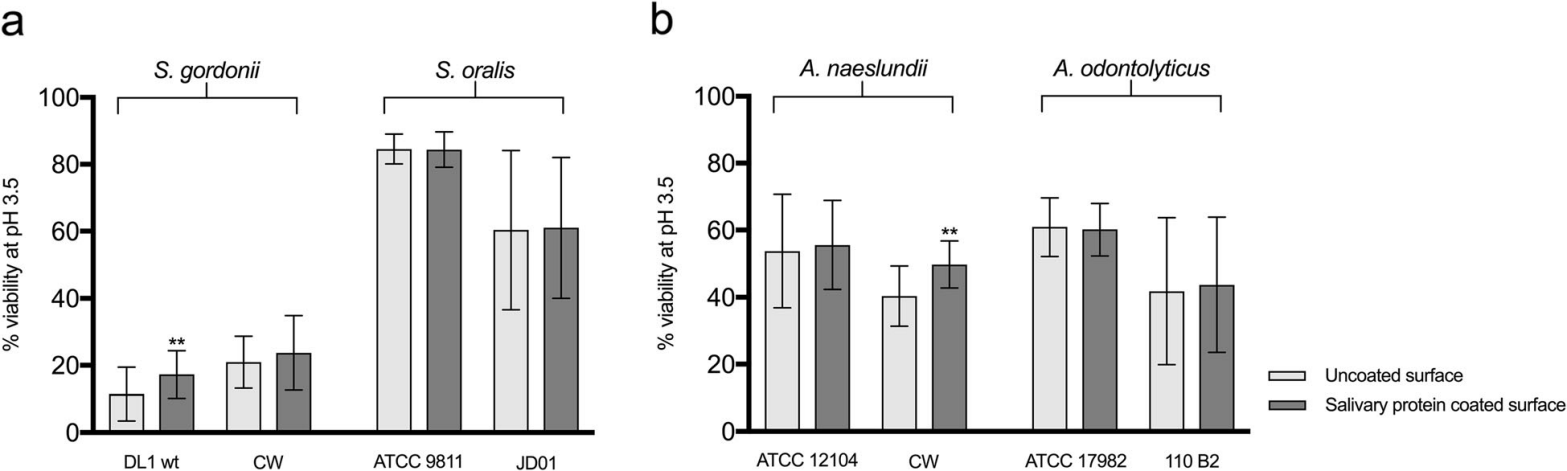
Comparison of the acid tolerance of biofilm cells of different streptococcal (**a**) and *Actinomyces* (**b**) strains on uncoated and salivary protein coated surfaces. Acid tolerance was evaluated after an acid challenge (pH 3.5) followed by LIVE/DEAD® BacLight™ staining and CLSM, and expressed as % viability. The graphs show the mean and standard deviation of three independent biological replicates. **P < 0.001

The surfaces to which bacteria adhere in the oral cavity are coated with a pellicle of saliva and therefore, we investigated whether salivary proteins could further enhance the surface-induced acid tolerance of the strains used in this study. Due to the fact that strains of *S. mutans* adhered poorly to salivary proteins (giving a surface area coverage of less than 1%), these were not investigated further. Neither of the strains of *S. oralis* showed an enhanced acid tolerance on adherence to a saliva-coating compared to uncoated surfaces. For *S. gordonii* however, the laboratory (DL1) and clinical strain (CW) showed 1.5-fold and 1.3-fold increases in acid tolerance respectively (Fig. 2a), and this change was significant for DL1 (*P* < 0.001). A similar picture was seen for the *Actinomyces* species where no enhancement was seen for the *A. odontolyticus* strains while a significant increase in acid tolerance was seen for the clinical strain of *A. naeslundii* (1.3-fold increase, *P <* 0.001), but not the laboratory strain (Fig. 2b). Thus, surface associated salivary proteins enhanced acid tolerance during biofilm formation for some strains of oral bacteria but not others.

### Development of an ATR

**Fig. 3:**
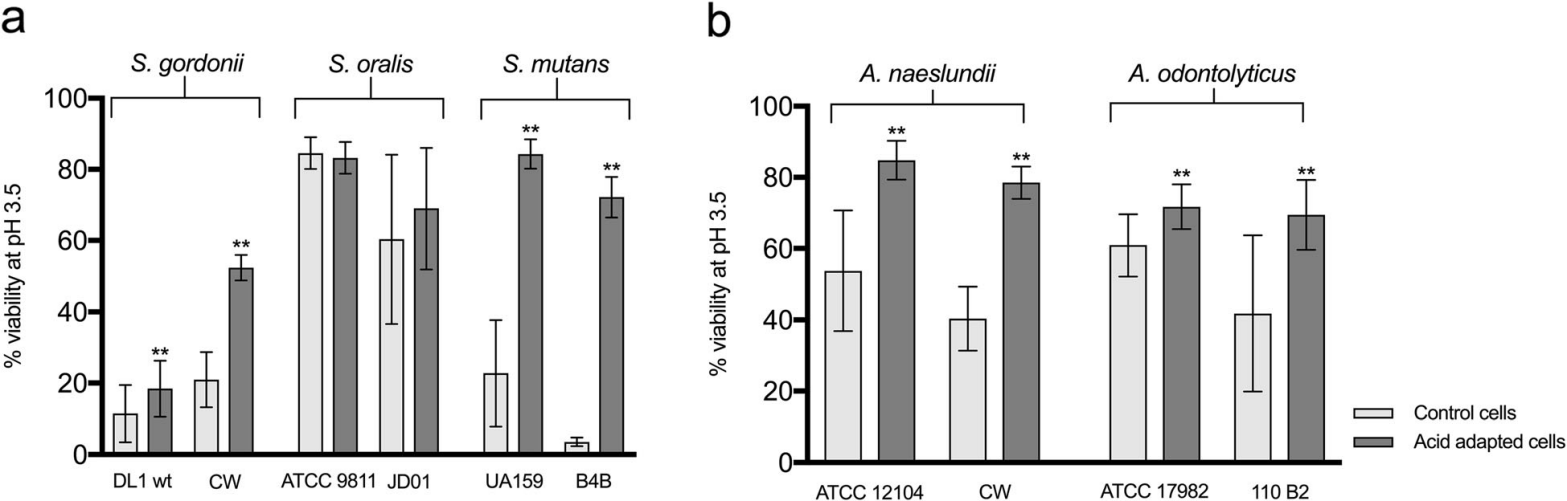
Ability of different streptococcal (**a**) and *Actinomyces* (**b**) strains to induce an ATR during exposure to an adaptation pH (pH 5.5). Control cells were kept at pH 7.5. Increased acid tolerance (indicating induction of an ATR) was evaluated after an acid challenge (pH 3.5) followed by LIVE/DEAD® BacLight™ staining and CLSM, and expressed as % viability. The graphs show the mean and standard deviation of three independent biological replicates. **P < 0.001

The ability of the bacterial strains to adjust to an acidic environment through induction of an ATR was determined by allowing them to adapt to pH 5.5 prior to the low pH challenge. Exposure to pH 5.5 did not in itself affect the viability of any of the strains as shown by staining with Baclight LIVE/DEAD (data not shown). With the exception of *S. oralis*, which was already highly acid tolerant, the acid tolerance increased significantly for all the strains of streptococci after a pre-exposure to pH 5.5 (*p* < 0.001, Fig. 3a). The largest difference was observed for the *S. mutans* strains, UA159 and B4B, which showed 3.7-fold and 18-fold increases respectively compared to non-adapted cells. Similarly, both strains of *S. gordonii* also showed enhanced acid tolerance after pre-exposure to pH 5.5 but the increase was greater for strain CW than DL1 (2.7-fold and 1.6-fold increases, respectively).

All the strains of *Actinomyces* showed small but significant (*P* < 0.001) increases in acid tolerance after pre-exposure to pH 5.5 (Fig. 3b). For both *A. naeslundii* and *A. odontolyticus*, the change in acid tolerance of the clinical strains (CW, 2-fold and 110B2, 1.7-fold increases) was somewhat higher than that for the laboratory strains (ATCC12104, 1.6-fold and NCTC17982, 1.2-fold increases). In summary, although *S. gordonii, A. naeslundii* and *A. odontolyticus* developed an ATR during biofilm formation, the ability of *S. mutans* to become acid tolerant through an ATR was greater than for the other strains tested.

### Effect of adhesion to salivary proteins on the ATR

**Fig. 4:**
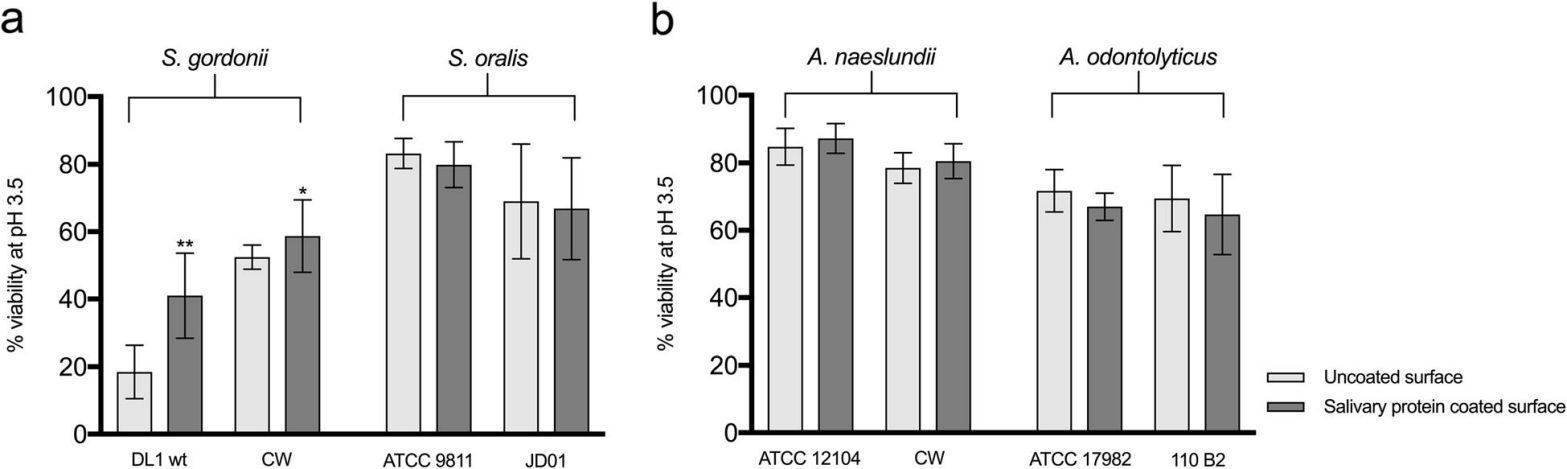
Effect of adhesion to salivary proteins on the ATR of different streptococcal (**a**) and *Actinomyces* (**b**) strains. Biofilm cells adhered to salivary protein coated or uncoated surfaces were exposed to an adaptation pH (pH 5.5). Increased acid tolerance (indicating induction of an ATR) was evaluated after an acid challenge (pH 3.5) followed by LIVE/DEAD® BacLight™ staining and CLSM, and expressed as % viability. The graphs show the mean and standard deviation of three independent biological replicates. *P < 0.05, **P < 0.001

To investigate the role of a salivary pellicle in development of an ATR, viability in response to the low pH challenge after adaptation at pH 5.5 was compared for each strain on an uncoated and a salivary protein-coated surface. For both the strains of *S. gordonii*, the presence of a salivary pellicle significantly increased ATR development in the biofilms compared to the uncoated surface (DL1, 2.3-fold increase, *P* < 0.001 and CW, 1.1-fold increase, *P* < 0.05). This effect was not seen for any of the other strains investigated (Fig. 4).

## Discussion

The ecological plaque hypothesis and its modifications state that in response to low pH, biofilm communities shift from a normal microbiota, with the majority of bacteria preferring to grow at around neutral pH, to a dysbiotic state associated with the selection and enrichment of acidogenic and aciduric species [13]. Since levels of *S. mutans* are often low, particularly at the early stages of a shift towards dysbiosis, other more abundant biofilm species including non-mutans streptococci and *Actinomyces spp* must play a significant role in initiating the development of an acid tolerant microbiota in caries [20]. Previously the acid tolerance and ability of some early colonizers to induce an ATR have been explored in planktonic cultures [21, 22], but how these bacteria behave in biofilms, and the effect of salivary proteins on acid tolerance development, has not been well characterized. Our data showed that the baseline acid tolerance (i.e. acid tolerance in the planktonic state) varied between the different species with, for instance, *S. oralis* showing high levels of survival following an acid challenge and *S. mutans* showing a rather low level. These data are in line with previous work where *S. sanguinis*, *S. oralis*, *S. mitis* and *S. mutans* showed differing degrees of acid tolerance [21], although in that study, the acid tolerance levels were reversed, with *S. mutans* being highly acid tolerant compared to *S. oralis.* Consistent with previous reports [29], all the *A. odontolyticus* and *A. naeslundii* strains showed some degree of acid tolerance, although the strains of *A. odontolyticus* showed a higher level of survival after acid challenge than those of *A. naeslundii*. The data revealed varying degrees of differences between the laboratory and the clinical strains for each species which is in agreement with published data showing heterogeneity in acid tolerance on the strain level [21, 28].

In the oral cavity bacteria are largely found as multi-species biofilms attached to saliva coated surfaces and it is generally accepted that biofilm cells are different to their planktonic counterparts [26, 30]. In *S. mutans* surface contact is known to enhance acid tolerance, and acid tolerance is further increased over time during biofilm maturation [28, 30]. It is therefore also possible that the acid tolerance in the species included in this study would increase as the biofilm grows older. In addition, genes involved in *S. mutans* response to acid stress are also known to be involved in biofilm formation suggesting a relation between these properties [31, 32]. In this study, for the strains exhibiting a low degree of inherent acid tolerance (including *S. mutans* UA 159), we observed a significant enhancement associated with adherence to a surface. During the early stages of biofilm formation, bacterial cells are known to be affected by electrochemical forces associated with the substrate and once adhered, they undergo physiological changes in gene and protein expression as well as morphology [33]. In addition, the metabolic state of the bacteria may change, with enhanced expression of glycolytic enzymes and increased ATP production [34, 35]. Studies on *Escherichia coli* have shown that the pH close to the surface may be lower than that of the surrounding liquid suggesting that local adaptation to a low pH environment may also contribute to an increase in acid tolerance [36]. Although the exact processes underlying the increase in acid tolerance seen on surface adherence of both the streptococci and *Actinomyces* strains are not known, some of the effect may, at least in part, be explained by the mechanisms mentioned above.

Since the surfaces to which bacteria adhere in the oral cavity are always covered with absorbed salivary proteins, the effect of adherence to a salivary pellicle was also investigated. In keeping with previous studies, the *S. mutans* strains adhered poorly to the salivary pellicle suggesting that although *S. mutans* is aggregated by salivary proteins in solution the binding epitopes are not exposed on these proteins when bound to a surface [37]. For the majority of the other bacteria which did bind, no additional effect was seen when salivary proteins were present compared to the uncoated surface. For *S. gordonii* (DL1) and *A. naeslundii* (CW) however, a significant increase in acid tolerance was seen on the saliva-coated surfaces suggesting that interaction between salivary proteins and bacterial adhesins may enhance the effect in these species. *S. gordonii* is known to express a range of surface adhesins with binding affinity for salivary proteins, including amylase and sialylated glycoproteins [38] and previously, binding to salivary amylase via the amylase-binding protein A (AbpA) resulted in an increased tolerance to low pH in this species [39]. Little is known about interactions between salivary proteins and *Actinomyces spp* but our data suggest that changes in gene expression resulting from signal transduction initiated by interaction between bacterial adhesins and salivary proteins may provide a mechanism whereby the ability of some bacteria to tolerate changes in pH is enhanced.

In supragingival plaque biofilms, bacteria are frequently exposed to periods of low pH due to generation of lactic acid through bacterial metabolism upon exposure to easily fermentable carbohydrates [40] and in some species exposure to pH 5.5 has been shown to induce an ATR [21, 22]. This phenomenon has been well characterized in the model organism *S. mutans* and includes alterations in bacterial physiology including changes in membrane permeability, increased ATPase activity and a lower pH optimum for glycolytic enzymes [41]. Overall, these changes better equip the bacteria to thrive in a lower pH environment. However, how the biofilm state affects the development of an ATR in bacteria other than *S. mutans* has not been fully investigated. In this study, the exposure to pH 5.5 for 2 h induced an ATR in all species except *S. oralis*. As expected, *S. mutans* UA159 displayed a substantial ATR and the most pronounced ATR was seen in clinical isolate *S. mutans* B4B with an 18-fold increase in acid tolerance. The ability to induce an ATR has been shown to differ between strains of oral bacteria and they have been categorized as “non-responders” which are unable to induce an ATR through to “strong responders”, which develop a robust ATR [42]. In biofilms, the *S. gordonii* strains behaved in a similar fashion to *S. mutans,* showing that these bacteria are able to induce an ATR at early stages of biofilm formation. In contrast*, S. oralis* which has been shown to induce an ATR in planktonic culture did not become more acid tolerant after adaptation in this study. However, a large population of the cells of the two strains used here were already inherently acid tolerant, a factor which may have masked their ability to induce an ATR. A similar phenomenon has been described previously by Takahashi and Yamada, where *S. sanguinis* displayed a high degree of inherent acid tolerance which did not increase further after adaptation [21]. *A. naeslundii* has previously been described as a non-responder but our findings show that both the strains used here have the ability to induce an ATR in biofilms. This phenomenon could be due to strain differences but may also be attributable to biofilm formation per se. *A. odontolyticus* responded in a similar manner and to the best of our knowledge this is the first time an ATR has been demonstrated for this species.

Surface associated salivary proteins did not appear to play a significant role in the development of an ATR for most of the species investigated, with the exception of *S. gordonii* where ATR development in biofilms was enhanced by the presence of a salivary pellicle. This finding strengthens the notion that interactions with salivary proteins are important in determining the physiology of this organism. Of particular interest in this context is the previous observation that binding of *S. gordonii* to salivary amylase leads to significant upregulation of genes involved in membrane fatty acid synthesis, since *S. mutans* is known to undergo changes in membrane fatty acid composition as part of the ATR [25, 43].

## Conclusions

In this study, the acid tolerance of a range of early oral colonizers varied and no consistent difference between the laboratory and clinical strains was observed. Many of the tested strains showed an increase in acid tolerance during biofilm formation and were able to induce an ATR in biofilms. For *S. gordonii*, the presence of a salivary pellicle enhanced both acid tolerance development and ATR induction. Overall, these findings suggest that colonizers of early oral biofilms can attain high levels of acid tolerance and that this can be reached in different ways. Physiological factors such as surface contact, the presence of a salivary pellicle and sensing of environmental pH, appear to contribute to the development of acid tolerance in early oral colonizers and thus be of importance for their role in the early stages of disease development in caries. However, while the studies presented here contribute significantly to our understanding of the role of early colonizers in acid tolerance development in biofilms, further investigations are needed to determine whether the phenomena identified here occur in the complex, multispecies communities found on the tooth surfaces in the oral cavity.

## Materials and methods

### Bacterial strains and media

The laboratory and clinical strains of the different bacterial species, as well as the media used for exponential phase growth are listed in Table 1. *S. mutans* UA159 previously shown to induce an ATR, was included for comparison. The acid tolerance and ATR experiments were performed using MM4 minimal medium containing 20 mM glucose, buffered with 40 mM phosphate/citrate buffer to pH 7.5, 5.5 or 3.5 [42]. Each strain was inoculated from blood agar to growth medium and incubated overnight at 37 °C in 5% CO_2_ in air. Aliquots of the overnight cultures were inoculated into growth medium and allowed to reach exponential growth phase (OD_600_ = 0.5–0.8) at 37 °C in 5% CO_2_ in air. The cells were then centrifuged at 5000 rpm, 5 °C for 5 min, washed twice with MM4 minimal medium buffered with 40 mM phosphate/citrate buffer at pH 7.5 and then resuspended in MM4 pH 7.5.

**Table 1:** Bacterial strains and exponential phase growth media used in this study

Bacterial species	Strain	Origin & Source	Growth medium
*Streptococcus gordonii*	DL1	ATCC 35105	Tryptone Yeast Extract
	CW	Dental plaque isolate	Tryptone Yeast Extract
*Streptococcus oralis*	9811	ATCC	Tryptone Yeast Extract
	JD01	Dental plaque isolate	Tryptone Yeast Extract
*Streptococcus mutans*	UA159	ATCC 700610	Todd-Hewitt Yeast Extract
	B4B	Dental plaque isolate	Todd-Hewitt Yeast Extract
*Actinomyces naeslundii*	12104	ATCC	Todd-Hewitt Broth
	CW	Dental plaque isolate	Todd-Hewitt Broth
*Actinomyces odontolyticus*	17982	NCTC	Todd-Hewitt Broth
	110 B2	Dental plaque isolate	Todd-Hewitt Broth

### Preparation of salivary proteins and coating of flow-cells

Unstimulated whole saliva was collected from 10 healthy volunteers aged 25–65, with no signs of active oral disease. Collections were made in the early morning and subjects asked to avoid eating and drinking for at least an hour prior to the saliva sampling. All volunteers gave written, informed consent and ethical approval was obtained from the human subject review committee at The Faculty of Odontology, Malmö University, Sweden (registration number OD2013/111). Subjects drooled for 30 min into a tube kept on ice after which the samples were pooled, mixed with an equal volume of ice-cold 0.2 M NaCl solution, and gently stirred overnight at 4 °C. The whole saliva sample was then centrifuged at 4400 *g* for 30 min at 4 °C in a Beckman Coulter Avanti J-E centrifuge in order to remove large particulate matter and the supernatant then subjected to cesium chloride (CsCl) density gradient centrifugation [7]. Briefly, CsCl was added to give a starting density of 1.45 g/ml and the sample centrifuged at 36000 rpm for 96 h at 15 °C, in a Beckman Coulter Optima LE-80 K equipped with a 50.2Ti rotor. After centrifugation, the fraction with a density greater than 1.59 g/ml (containing primarily DNA and bacteria) was discarded and the remaining sample dialyzed to equilibrium against artificial saliva buffer, ASB (15.6 mM KCl, 2.6 mM KH_2_PO_4_, 0.2 mM MgCl_2_, 2.6 mM Na_2_HPO_4_, 10 mM NaCl, 4.4 mM NH_4_Cl, pH 6.8) [44] using Spectra/Por dialysis membrane with a cut-off of 3500 Da. The sample of whole “native” saliva with the bacteria removed, containing all the major salivary proteins, was kept at − 20 °C until use. To obtain a salivary protein coated surface for biofilm formation, the channels of Ibidi® μ-slide VI Ibi-treat flow-cells (Ibidi GmbH, Gräelfing, Germany) were coated overnight in a humid chamber at room temperature with whole native saliva in the presence of 1 mM CaCl_2_. The channels of the flow cells were rinsed with 10 mM PBS pH 7.2 before use.

### Acid tolerance of different early oral colonizers

The acid tolerance of the bacteria used in this study was assessed by measuring their viability after exposure to a low pH challenge (pH 3.5) in planktonic culture [28]. Cells in exponential growth phase were suspended in MM4, pH 7.5 and incubated at 37 °C in 5% CO_2_ for 2 h. The cells were then washed with MM4 pH 3.5, centrifuged at 5 °C, 5000 rpm for 5 min and the pellets resuspended in MM4 pH 3.5 prior to incubation at 37 °C in 5% CO_2_ for 30 min. Control cells were treated in the same manner, but washed and resuspended in MM4, pH 7.5. Finally, the cells were centrifuged at 5 °C, 5000 rpm for 5 min and pellets resuspended in LIVE/DEAD® BacLight™ solution (Molecular Probes, Eugene, Oreg., USA) prepared according to the supplier’s instructions. Aliquots were introduced into Ibidi® μ-slide VI Ibi-treat flow-cells which were then centrifuged gently at 5 °C, 1000 rpm for 1 min. Flow-cells were then viewed with confocal laser scanning microscopy (CLSM) using a Nikon Eclipse TE2000 microscope (Nikon Corp., Tokyo, Japan) with an Ar laser (488 nm laser excitation). Images were acquired with a Photometrics Prime 95B camera using Nikon NIS-Elements software. Ten randomly selected images from each experimental condition were saved for image analysis.

### Effect of early biofilm formation on acid tolerance

**Fig. 5:**
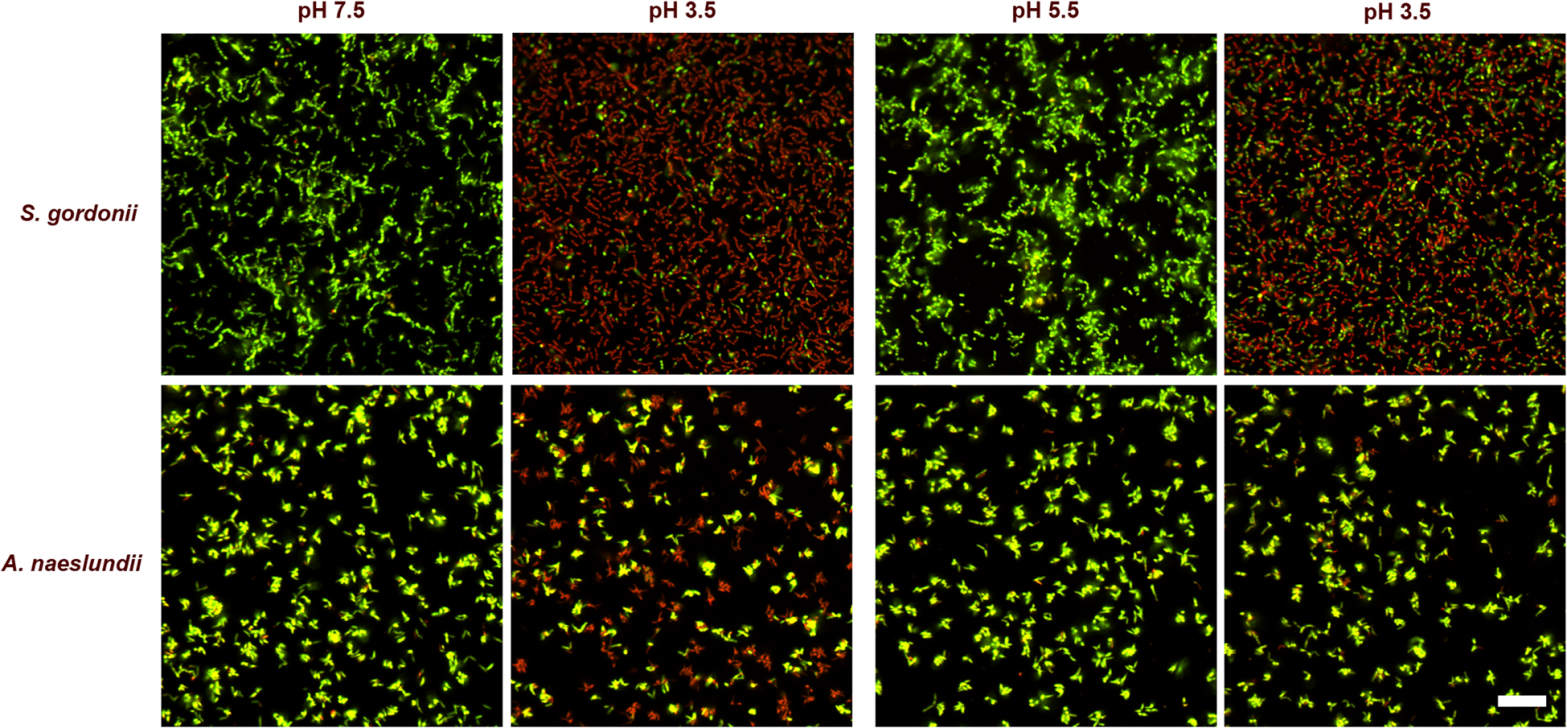
Example images of LIVE/DEAD® BacLight™ stained cells of *S. gordonii* CW and *A. naeslundii* CW viewed with CLSM. Control cells were kept at pH 7.5 and then exposed to acid challenge (pH 3.5). For ATR induction, cells were exposed to an adaptation pH (pH 5.5) for 2 h prior to exposure to pH 3.5. Viable cells (acid tolerant) appear green while dead cells (non-acid tolerant) appear red. Scale bar shows 20 μm

Biofilms were prepared by introducing aliquots of exponential growth phase cells of each bacterial strain in MM4 pH 7.5 into μ-slide VI Ibi-treat flow-cells, either uncoated or coated with salivary proteins (see above). The flow-cells were then incubated in a humid chamber in 5% CO_2_ in air at 37 °C for 2 h in order for the cells to adhere to the surface. The channels of the flow-cells were then washed three times with MM4 pH 7.5 and the biofilm cells incubated in MM4 pH 7.5 as described above for an additional 2 h. Finally, the cells were washed three times with MM4 pH 3.5 and exposed to the low pH challenge for 30 min. Excess fluid was removed and LIVE/DEAD® BacLight™ solution added to each lane. Control cells were kept at pH 7.5, but otherwise treated in the same manner. Flow-cells were examined using CLSM as described above and 10 random images from each experimental condition were saved for image analysis (Fig. 5).

### Acid tolerance response of biofilm cells

The ability of the two-hour biofilm cells to induce an ATR was evaluated by pre-exposing them to pH 5.5 prior to the low pH challenge (pH 3.5). Following 2 h of biofilm formation in the μ-slide VI Ibi-treat flow-cells, the bacteria were washed three times with MM4 pH 5.5 and then incubated in MM4 pH 5.5 in a humid chamber in CO_2_ at 37 °C for 2 h. The flow-cells were then washed with MM4 pH 3.5 three times and incubated for 30 min. Excess fluid was removed before LIVE/DEAD® BacLight™ solution was added to each channel. Control cells were kept at pH 7.5 and 5.5 respectively, but otherwise treated in the same manner. The flow cells were examined using CLSM and 10 images from each experimental condition saved for image analysis (Fig. 5).

### Image analysis

Image analysis determining the surface coverage and percentage viability was performed using software *bio*Image_L [45]. Briefly, bioImage_L applies a colour segmentation routine that automatically segments the colour image into individual pseudo channels, and the areas and percentages of each identified colour subpopulation are calculated and presented. Viability was assessed as viable cell count (green cells) standardized against total bacterial counts [green + red cells (dead cells)].

### Statistics

All experiments were undertaken in triplicate using independent biological replicates and all 10 images from each experiment were included in the statistical analysis using the Mann-Whitney U test (software GraphPad Prism, San Diego, USA). *P*-values of less than 0.05 were considered statistically significant.

## Data Availability

The datasets used and/or analysed during the current study are available from the corresponding author on reasonable request.
